# Single-Nucleotide Polymorphisms Associated with Skin Naphthyl–Keratin Adduct Levels in Workers Exposed to Naphthalene

**DOI:** 10.1289/ehp.1104304

**Published:** 2012-03-05

**Authors:** Rong Jiang, John E. French, Vandy P. Stober, Juei-Chuan C. Kang-Sickel, Fei Zou, Leena A. Nylander-French

**Affiliations:** 1 Department of Environmental Sciences and Engineering, Gillings School of Global Public Health, University of North Carolina–Chapel Hill, Chapel Hill, North Carolina, USA; 2 National Toxicology Program, National Institute of Environmental Health Sciences, National Institutes of Health, Department of Health and Human Services, Research Triangle Park, North Carolina, USA; 3 Department of Biostatistics, Gillings School of Global Public Health, University of North Carolina–Chapel Hill, Chapel Hill, North Carolina, USA

**Keywords:** biomarker, candidate-gene analysis, exposure assessment, genome-wide analysis, jet fuel, naphthalene, relative contribution, single-nucleotide polymorphism, skin keratin adduct

## Abstract

Background: Individual genetic variation that results in differences in systemic response to xenobiotic exposure is not accounted for as a predictor of outcome in current exposure assessment models.

Objective: We developed a strategy to investigate individual differences in single-nucleotide polymorphisms (SNPs) as genetic markers associated with naphthyl–keratin adduct (NKA) levels measured in the skin of workers exposed to naphthalene.

Methods: The SNP-association analysis was conducted in PLINK using candidate-gene analysis and genome-wide analysis. We identified significant SNP–NKA associations and investigated the potential impact of these SNPs along with personal and workplace factors on NKA levels using a multiple linear regression model and the Pratt index.

Results: In candidate-gene analysis, a SNP (rs4852279) located near the *CYP26B1* gene contributed to the 2-naphthyl–keratin adduct (2NKA) level. In the multiple linear regression model, the SNP rs4852279, dermal exposure, exposure time, task replacing foam, age, and ethnicity all were significant predictors of 2NKA level. In genome-wide analysis, no single SNP reached genome-wide significance for NKA levels (all *p* ≥ 1.05 × 10^–5^). Pathway and network analyses of SNPs associated with NKA levels were predicted to be involved in the regulation of cellular processes and homeostasis.

Conclusions: These results provide evidence that a quantitative biomarker can be used as an intermediate phenotype when investigating the association between genetic markers and exposure–dose relationship in a small, well-characterized exposed worker population.

Significant individual variation exists in systemic responses to xenobiotic exposures that may be due to individual differences in environmentally responsive genes and pathways (Christiani et al. 2008). Sources of genetic variation include single-nucleotide polymorphisms (SNPs) that alter mRNA coding or expression (Hinds et al. 2006; McCarroll et al. 2008), copy number variation (Conrad et al. 2010), and altered patterns of CpG methylation and histone modifications that affect gene regulation and expression (Boks et al. 2009; Gibbs et al. 2010). Consequently, interindividual genetic differences and other host factors can affect resistance or susceptibility to toxicity and disease (Adeyemo et al. 2009; de Geus et al. 2005; Glinskii et al. 2009). However, our knowledge of the basis for individual differences in exposure, toxicity, and associated health effects and of the value of including information on individual genetic variation in exposure assessment models is limited.

Genetic association studies between individual genotypes and phenotypes within a population can be used to discover genetic variants that modulate exposure-related phenotypes. Most genetic association studies have evaluated disease end points using case–control study design (Collins 2009; Cordell and Clayton 2005; Suh and Vijg 2005; Zondervan and Cardon 2007). Recent studies suggest that mapping intermediate steps in disease processes, that is, quantitative intermediate phenotypes (e.g., clinical traits, metabolites, gene transcript expression levels), may be more informative than estimating associations with discrete binary case–control disease status (Chen et al. 2008; Emilsson et al. 2008; Keller et al. 2008) because intermediate phenotypes may be more directly influenced by genetic variation. Instead of using binary outcomes, individual differences in systemic exposure levels (parent compound or metabolite) in a population of concurrently exposed individuals can serve as intermediate phenotype when investigating relationships between dose–response and individual susceptibility to toxicity and disease. Treating a biomarker of exposure as an intermediate phenotype could reduce bias due to exposure misclassification in case–control status and increases power because dichotomization leads to loss of information, especially in exposure assessment studies.

Interactions between genetic and environmental factors are critical to toxicity and disease risk (Christiani et al. 2008; Vineis et al. 2009; Vlaanderen et al. 2010). Exposure assessment and epidemiology studies that use biomarkers of exposure and/or effect to reveal the exposure–disease relationships must account for interindividual variation. Identifying variation associated with individual genetic markers, such as SNPs as variance components, has the potential to provide mechanistic insight into toxicity and the etiology of disease and to inform efforts to set exposure limits and required interventions to reduce risk in susceptible individuals.

Previously, we demonstrated that fuel-cell maintenance personnel who performed fuel-tank entry tasks had higher exposure to naphthalene via both dermal (Chao et al. 2005) and inhalation routes (Egeghy et al. 2003; Serdar et al. 2004) than did personnel who did not enter the fuel tanks. We also demonstrated that skin naphthyl–keratin adducts (NKAs) can be used as biomarkers for jet fuel exposure (Kang-Sickel et al. 2010, 2011). Our goal was to develop an exposure assessment strategy to resolve the impact of individual genetic variation, along with personal and workplace factors, on measured biomarker levels in a small, well-characterized worker population exposed to naphthalene in a complex mixture (jet fuel). We also investigated the biological relevance of significant SNP-associated genes using pathway and network analysis to evaluate the impact and plausibility of individual variants on the observed biomarker level.

## Methods

*Study population.* Exposure data were available for U.S. Air Force (USAF) personnel exposed to jet propulsion fuel 8 (JP-8) at six USAF bases in the continental United States (Chao et al. 2005, 2006; Egeghy et al. 2003). Participants were recruited from active-duty personnel who routinely worked with or were exposed to JP-8, and all participants provided informed consent. Approval for human subject use was obtained from the institutional review board for the USAF and for each of the participating institutions, and the study complied with all applicable U.S. requirements and regulations.

Of the 339 workers enrolled in the overall project, 105 USAF fuel-cell maintenance workers who were monitored for both dermal and inhalation exposure were included in the present study. Questionnaires were collected after a work shift to obtain data on demographic factors, including job tasks, use of personal protective equipment, smoking status, and other work-related factors. A diary for each worker was recorded during the surveys by the research staff, including detailed information on work tasks and durations. Participants included those who entered the plane’s fuel cell and performed maintenance tasks, including tank door, bolt, and/or foam removal; foam replacement; fuel-tank cleaning; depuddling; and repairs and inspections (referred to as entrants). We also included attendants and runners who worked outside the fuel tank to assist entrants and other field workers who performed maintenance with occasional contact with JP-8 fuel.

*Measurements of dermal exposure.* We used naphthalene as an exposure marker for jet fuel (Chao et al. 2005, 2006). Dermal exposure to naphthalene was quantified using samples from three exposed body regions of each worker after the work shift as described previously by Chao et al. (2005). Samples were collected using adhesive tape strips (Cover-Roll™; Beiersdorf AG, Hamburg, Germany).

*Skin naphthyl–keratin adduct sampling and analysis.* Tape-strip samples were collected from each worker as described for dermal naphthalene exposure and quantified for four different NKAs using an enzyme-linked immunosorbent assay (Kang-Sickel et al. 2008, 2010). The total 1-naphthyl–keratin adduct (1NKA) levels were calculated by summing the 1-naphthyl-keratin-1 and 1-naphthyl-keratin-10 levels, whereas total 2-naphthyl–keratin adduct levels (2NKA) were calculated by summing the 2-naphthyl-keratin-1 and 2-naphthyl-keratin-10 levels. The total NKA (TNKA) level for each worker was calculated by summing the four keratin-normalized adduct levels.

*Genotyping.* Genomic DNA isolated from peripheral blood was used to genotype each worker using the GeneChip® Human Mapping 250k *Sty*I SNP array (Affymetrix, Santa Clara, CA) according to the manufacturer’s protocol.

Among 105 individuals, 3 were excluded because of low genotype rate (missing genotype rate > 10% per person). In addition, 28,445 markers with low genotype rate (missing genotype per SNP > 10%), 18,744 SNPs with < 1% minor allele frequency (MAF < 0.01), and 4,840 SNPs with Hardy-Weinberg departure (*p* < 0.001) along with the X chromosome were also excluded. The average call rate was 98.1%.

*Statistical analyses.* Descriptive statistics were derived using SAS (version 9.2; SAS Institute Inc., Cary, NC). We performed association analyses between SNP alleles and total 1NKA levels, total 2-naphthyl–keratin adduct (2NKA) levels, and total NKA (TNKA; the sum of 1NKA and 2NKA) levels using PLINK, version 1.06 (Purcell et al. 2007). PLINK is a free, open-source whole genome-wide association analysis tool set that allows the use of either asymptotic (likelihood ratio test and Wald test) or empirical significance values (permutation). PLINK also allows adjustment for multiple covariates when testing for quantitative SNP–NKA level association. All NKA levels and the measured naphthalene dermal exposure levels were log-transformed. An additive model was used, and each SNP genotype was coded as 0, 1, or 2 for noncarriers, heterozygous carriers, and homozygous carriers of the minor allele, respectively; in this way, the mean value of the biomarker level increases with the variant allele frequency. We investigated SNP associations using candidate-gene analysis (CGA) and genome-wide analysis (GWA) approaches, controlling for other important covariates. SNPs highly associated with each corresponding adduct level were selected as potential independent variables in the exposure assessment model to estimate interindividual genetic contribution relative to other significant personal and workplace covariates.

Determination of covariates. SNP association analysis was performed by controlling for personal and workplace factors that were significant predictors of the measured levels of NKAs. To accomplish this, we developed multiple linear regression models (MLRMs) using SAS, which included dermal naphthalene level and significant personal and workplace factors (α = 0.1). The general form of a MLRM is


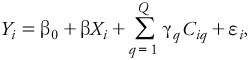
[1]

where *Y_i_* represents the natural log of the NKA level of the *i*th worker; *X_i_* represents the natural log of the dermal naphthalene level for the *i*th worker; *C_iq_* represents the *q*th covariate value for the *i*th worker (i.e., personal and workplace factors); β and γ*_q_* represent the regression coefficients for the dermal naphthalene level and *q*th covariate, respectively; β_0_ is the intercept; and ε*_i_* is the random error for the *i*th worker.

We used STEPWISE model selection in PROC REG (SAS) to determine regression model 1, using dermal naphthalene level and other covariates as potential independent variables. Only naphthalene through dermal exposure can induce formation of NKAs in the skin (i.e., these adducts are route-specific indicators of exposure; inhalation exposure will not contribute to NKA formation). Possible collinearity problems were investigated using eigenvalue analyses and variance inflation factors. Possible outliers were examined using studentized residuals. Residual analysis was performed to check if the fitted models met all assumptions.

SNP association analyses. Because personal and workplace factors can influence association between SNPs and skin NKA levels, associations were estimated by controlling for the covariates determined in model 1. PLINK allows for multiple covariates when testing for a quantitative trait using linear models. The general form of the linear model in PLINK is


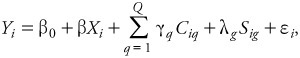
[2]

where *S_ig_* represents the *g*th tested SNP type for the *i*th worker as individual genetic data, and λ*_g_* represents the coefficient for *g*th SNP. Everything else remained as in model 1. One SNP association was tested at a time controlling for covariates using model 2.

We first conducted a focused NKA CGA to identify SNPs associated with specific genes. Candidate genes were selected based on published known or suspected functional and/or regulatory roles in the metabolism and toxicokinetics of naphthalene and/or polyaromatic hydrocarbons (PAHs) [for the list of 35 candidate genes, see Supplemental Material, Table 1 (http://dx.doi.org/10.1289/ehp.1104304)]. SNPs related to candidate genes were obtained from the Affymetrix database (Affymetrix 2009). Because SNPs may be associated with more than one candidate gene, and some SNPs were excluded by the data-cleaning procedure, 498 SNPs were used in the CGAs. Each SNP genotype was individually tested for its association with NKA level under model 2.

**Table 1:** Regression models for skin NKA levels measured in fuel-cell maintenance workers using GWA.

Adduct/predictor	Parameter estimate	SE	p-Value	Relative contribution (%)
1NKA (n = 90; R2 = 0.665)								
Intercept		–0.821		0.297		0.007		
Exposure time		0.002		0.001		0.014		6.71
Dermal exposure		–0.029		0.014		0.037		0.34
Replace foam		0.325		0.068		< 0.0001		22.49
Age		–0.016		0.006		0.009		3.56
Smoking		0.156		0.059		0.010		2.58
rs33977056		0.141		0.047		0.003		13.36
rs2000753		–0.177		0.056		0.002		11.63
rs3799570		0.161		0.058		0.007		10.49
rs1329508		0.103		0.048		0.035		10.10
rs2286321		0.127		0.052		0.016		9.71
rs10413028		0.145		0.057		0.013		9.02
2NKA (n = 87; R2 = 0.721)								
Intercept		0.783		0.239		0.002		
Exposure time		0.004		0.001		< 0.0001		28.65
Dermal exposure		–0.061		0.013		< 0.0001		3.90
Replace foam		0.105		0.060		0.084		4.90
Age		–0.016		0.005		0.003		4.66
Ethnicity		–0.156		0.069		0.026		2.74
rs11251918		0.196		0.047		< 0.0001		14.10
rs9835822		–0.135		0.040		0.001		13.97
rs6486693		–0.107		0.038		0.007		11.40
rs4971689		0.159		0.043		0.0004		11.37
rs9295589		–0.098		0.047		0.039		4.30
TNKA (n = 83; R2 = 0.729)								
Intercept		0.831		0.238		0.001		
Exposure time		0.005		0.001		< 0.0001		39.65
Dermal exposure		–0.066		0.012		< 0.0001		2.13
Replace foam		0.100		0.055		0.075		4.87
Age		–0.021		0.005		< 0.0001		5.52
Ethnicity		–0.192		0.064		0.004		2.59
rs1962392		–0.135		0.036		0.0004		12.03
rs11251918		0.171		0.044		0.0002		11.50
rs4971689		0.145		0.040		0.001		10.43
rs3799570		0.128		0.043		0.004		6.17
rs9295589		–0.122		0.042		0.004		5.11

GWA was performed to identify SNPs significantly associated with NKA levels without *a priori* evidence of potential association, regardless of mechanism. Specifically, each SNP genotype was tested for association across individual genomes using model 2. To adjust for multiple comparisons, empirical *p*-values were calculated using the max(*T*) permutation approach implemented in PLINK for both CGA and GWA (*n* = 10,000 permutations each). The overall level of significance was 0.1. Genome-wide *p*-value against SNP physical position (by chromosome) genome plots were generated using the gap package in R, version 2.7.2 (Zhao 2007). The goal was to identify SNPs that were most significantly associated with skin NKA levels for inclusion in a final exposure model that incorporated other predictors of exposure.

Exposure modeling. To investigate the genetic predictors of population variance in individual NKA biomarker levels, we estimated the contribution of individual SNP markers in addition to personal (e.g., dermal exposure, personal protection, smoking) and workplace (e.g., job, task, work environment) factors. The MLRMs were developed using SAS, and the significance was evaluated at α = 0.1 because of the number of uncontrolled variables affecting biomonitoring. The general form of MLRM was modified to


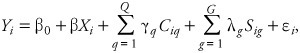
[3]

where *G* denotes the number of SNPs identified as significant through CGA or GWA and *S_ig_* is the genotype of the *g*th significant SNP with corresponding coefficient λ*_g_*. All other parameters are as previously defined. Significant SNPs and covariates were selected as potential independent variables. STEPWISE selection was used in PROC REG to determine regression model 3, and model analysis was performed using the methods described for model 1.

The relative contribution of each predictor in the final regression models was determined by its proportionate contribution to the regression model *R*^2^ using the Pratt index, which is the product of the estimated standardized regression coefficient and the simple correlation between that predictor and the outcome variable (Chao et al. 2008; Pratt 1987). The relative contribution considered in this study was the dispersion importance (i.e., the proportion of the variance in the outcome variable accounted for by each predictor in the regression model).

Bioinformatic analysis of highly associated SNPs and network interactions. We investigated all SNPs that were significantly associated with NKA levels; the curated sequence identification, location, and gene ontology were established using Entrez Gene (http://www.ncbi.nlm.nih.gov/snp) (Maglott et al. 2005), Ensembl BioMart (http://www.biomart.org) (Flicek et al. 2011), and/or UCSC Genome Browser (http://genome.ucsc.edu) (Fujita et al. 2011). Interactions between genes related to SNPs that predicted NKA levels were tested for statistical significance by MetaCore™ integrated knowledge database (http://www.genego.com/metacore.php) and software suite for network/pathway analysis using the proprietary database of hand-curated peer-reviewed literature and statistical analysis of network interactions (Brennan et al. 2009; Chang 2009; Thomson Reuters 2010). The predicted subnetworks associated with the highest-ranked *p*-value for SNP-TNKA were determined using the highest trust set using the most probable linear binary protein interactions (i.e., verified through experimental validation). Highly relevant identified gene functions were corroborated by further literature analysis of the predicted associations [formula available from MetaCore™ (Thomson Reuters 2010)].

## Results

*Study population and exposure measurements.* After data cleaning, 184,153 SNPs and 102 workers were available for association analyses. The workers included 94 males (92.2%) and 8 females (7.8%), of whom 89 were Caucasians (87.3%), 13 were non-Caucasians (African American, Hispanic, or Asian; 12.7%), and 45 were smokers (44.1%). The mean ± SD age of the workers was 24.6 ± 5.0 years, and ages ranged from 18 to 40 years.

The geometric mean (GM) and geometric SD (GSD) of the dermal naphthalene levels were 1,556 and 8.6 ng/m^2^, with a range of 100 ng/m^2^ to 5,090 μg/m^2^. Both 1NKA and 2NKA were detected in the tape-strip samples at levels of 0.27–6.4 pmol/µg keratin. The GM (GSD) for 1NKA, 2NKA, and TNKA levels were 0.7 (1.5), 2.1 (1.5), and 2.8 (1.5) pmol/µg keratin, respectively. The 1NKA GM level was significantly different (*p* < 0.0001) from that of 2NKA. Dermal exposure, exposure time, age, ethnicity, and the replacing foam task were significantly associated with 2NKA and TNKA levels [all, *p* < 0.094; see Supplemental Material, Table 2 (http://dx.doi.org/10.1289/ehp.1104304)]. In addition, smoking, but not ethnicity, was significantly associated with 1NKA level (*p* < 0.086; see Supplemental Material, Table 2). Therefore, the final CGA and GWA models for 2NKA and TNKA levels included age, exposure time, dermal exposure, ethnicity, and the replacing foam task, whereas ethnicity was replaced with smoking status in the final models for 1NKA. Because of missing exposure-time information for two subjects, 100 subjects were included in the CGA and GWA models.

**Table 2:** SNP variants and genes associated with the skin NKA levels and the significant covariates affecting naphthalene exposure in the fuel-cell maintenance workers as identified by GWA and multiple linear regression models.

Chromosome	Build 37.1 position	SNP	Alleles	Associated genes	MAF	GWA p-value (× 10–5)
2p14		63597786		rs33977056		A/G		WDPCP		0.208		2.24
2p16.3		50775436		rs4971689		C/G		NRXN1		0.292		2.76
2q37		241653539		rs2286321		A/G		SNED1		0.222		4.19
3p13.1		109214614		rs9835822		C/T		BBX, CD47		0.293		1.83
6p22		23553309		rs9295589		A/G		RPL6P18, NRSN1		0.223		6.88
6q26		162672040		rs2000753		C/T		PARK2		0.266		2.79
6q27		166950610		rs3799570		C/T		RPS6KA2		0.162		3.23
8p23.1		10299783		rs1962392		C/G		MSRA		0.302		5.80
10p15		3535288		rs11251918		G/T		PITRM1, KLF6		0.138		5.29
10q25.1		110184413		rs1329508		A/G		SORCS, ADD3		0.358		7.26
12q24.32		127551455		rs6486693		C/T		TMEM132C		0.470		9.03
19q12		27148774		rs10413028		A/G		ZNF536, TAF2GL		0.172		5.34
Abbreviations: BBX, bobby sox homolog (Drosophila); WDPCP, WD repeat containing planar cell polarity effector; PITRM1, pitrilysin metallopeptidase 1; RPL6P18, ribosomal protein L6 pseudogene 18; SNED1, sushi, nidogen and EGF-like domains 1; SORCS, sortilin-related VPS10 domain containing receptor 3; TAF2GL, TAF9 RNA polymerase II, TATA box binding protein (TBP)-associated factor, 32kDa pseudogene 3; TMEM132C, transmembrane protein 132C; ZNF536, zinc finger protein 536.												

*Candidate-gene analysis.* One SNP (rs4852279; MAF = 0.451) related to cytochrome P450, family 26, subfamily B, polypeptide 1 (*CYP26B1*) was significantly associated with 2NKA level (permutation adjusted *p* = 0.0449). No SNP associated with any candidate gene [see Supplemental Material, Table 2 (http://dx.doi.org/10.1289/ehp.1104304)] was significantly associated with 1NKA or TNKA levels. In the MLRM model, SNP rs4852279 (associated with *CYP26B1*) and five covariates (i.e., dermal exposure, exposure time, task replacing foam, age, and ethnicity) were significant predictors of 2NKA level.

*Genome-wide analysis.* No single SNP reached genome-wide significance for association with NKA biomarkers (at α = 0.1/184,000, *p* = 5.4 × 10^–7^) [see Supplemental Material, Figure 1 (http://dx.doi.org/10.1289/ehp.1104304)]. The SNPs that were most significant for 1NKA, 2NKA, and TNKA levels were rs2286321 (*p* = 1.20 × 10^–5^), rs11889897 (*p* = 1.05 × 10^–5^), and rs4971689 (*p* = 1.75 × 10^–5^), respectively. The locations and the patterns of significant SNPs associated with skin NKA levels varied for each adduct type.

**Figure 1:**
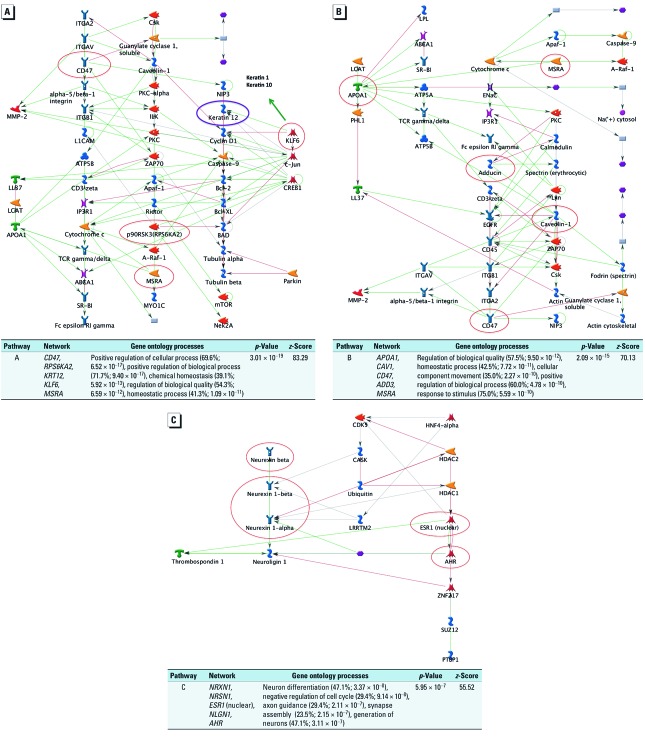
Three predicted MetaCore™ network interaction pathways (*A–C*) from 12 polymorphic SNP genetic markers associated with the skin NKA levels measured in the fuel-cell maintenance workers. Abbreviations: *AHR*, aryl hydrocarbon receptor; *APOA1*, apolipoprotein A-1; *CAV1*, caveolin 1, caveolae protein, 22kDa; *ESR1*, estrogen receptor 1; *KRT12*, keratin 12; *NLGN1*, neuroligin 1.

Ten SNPs with the lowest unadjusted *p*-values for each of the three NKAs were tested, along with corresponding significant covariates, to estimate their effect and relative contributions to the measured level of each NKA using MLRM. In the final models, six, five, and five SNPs were retained as significant predictors for 1NKA, 2NKA, and TNKA, respectively (Table 1). These final models explained 66%, 72%, and 73% of the total variation in the 1NKA, 2NKA, and TNKA levels, respectively, for the covariates tested.

*Bioinformatics.* Of the 16 SNPs highly associated with NKA levels identified by GWA and MLRM analyses, 4 were associated with the same sequence, leaving 12 SNPs that were unique genic or intergenic sequences (Table 2). Eight of these 12 highly associated SNPs were within or proximate to known genes {*ADD3* [adducin 3 (gamma)], *CD47* (CD47 molecule), *RPS6KA2* (ribosomal protein S6 kinase, 90kDa, polypeptide 2), *KLF6* (Kruppel-like factor 6), *PARK2* [parkinson protein 2, E3 ubiquitin protein ligase (parkin)], *MSRA* (methionine sulfoxide reductase A), *NRSN1* (neurensin 1), and *NRXN1* (neurexin 1)} that likely interact through three networks (Figure 1) predicted by multiple binary protein–protein interactions as statistically associated in the curated published literature (Brennan et al. 2009; Chang 2009; Thomson Reuters 2010). None of the three predicted subnetworks were associated with any known canonical pathway.

The first predicted network involves *CD47*, *RPS6KA2*, *KLF6*, and *MSRA* (Figure 1A), which are associated with the positive regulation of cellular and biological processes, chemical homeostasis, regulation of biological quality, and homeostasis (*p* = 3.01 × 10^–19^). In the second predicted pathway (Figure 1B), *ADD3*, along with *MSRA* and *CD47*, may also interact independently with *APOA1* (apolipoprotein A-1) and *CAV1* (caveolin 1, caveolae protein, 22kDa) to regulate biological quality, homeostasis, cellular components of movement, positive regulation of biological process, and response to stimuli (*p* = 2.09 × 10^–15^). In the third pathway (Figure 1C), *NRXN1* and *NRSN1* are associated with neuron differentiation, negative regulation of cell cycle, axon guidance, synapse assembly, and generation of neurons in the published literature.

The remaining four SNPs are in or proximate to genes {*SNED1* (sushi, nidogen and EGF-like domains 1), *TMEM132C* (transmembrane protein 132C), *WDPCP* (WD repeat containing planar cell polarity effector), *ZNF536* (zinc finger protein 536), and *TAF2GL* [TAF9 RNA polymerase II, TATA box binding protein (TBP)-associated factor, 32kDa pseudogene 3; *TAF9P3*]} that were not predicted from the curated literature to interact within any predicted networks, and none has known relevant function in skin or appears to be a structural protein integral to cell or basement membranes. For detailed discussion of the bioinformatic analyses, see Supplemental Material, pp. 8–10 (http://dx.doi.org/10.1289/ehp.1104304).

## Discussion

An individual’s quantitative NKA level can be treated as an intermediate phenotype to provide increased power to detect quantitative associations in well-defined populations exposed to naphthalene. Our goal was to investigate and identify SNPs associated with NKA biomarker levels for use in exposure assessment models.

In the CGA approach, we identified one SNP related to *CYP26B1* that was significantly associated with 2NKA level. *CYP26B1* function is involved in the regulation of all-*trans*-retinoic acid (RA), which regulates epidermal proliferation and differentiation, keratinocyte proliferation, and epidermal hyperplasia (Giltaire et al. 2009). RA is the only recognized substrate of CYP26B1 detected in human skin (Ray et al. 1997), and it has been shown to inhibit *KRT10* (keratin 10) mRNA and protein expression in epidermal keratinocytes *in vitro* (Poumay et al. 1999). Variation in CYP26B1 expression may influence RA levels in the skin leading to differences in KRT10 protein expression and thus may affect the skin NKA levels observed in the present study.

The CGA approach may be limited because of probe selection in the Affymetrix *Sty*I array. Among 498 SNPs associated with candidate genes, only 9 were located within exons {*CYP1A2*, *ARNT2* (aryl-hydrocarbon receptor nuclear translocator 2), *EPHX1* [epoxide hydrolase 1, microsomal (xenobiotic)], *EPHX2*, *CYP26B1*, *GSTM3* (glutathione *S*-transferase mu 3 (brain)], and *CYP4B1*} and 87 were located within introns. The remaining SNPs were within intergenic regions, distal to either 5´ or 3´ of potential target genes. Even the SNP that is significantly associated with 2NKA level (rs4852279) is about 110 kb 5´ to the *CYP26B1* gene body. Furthermore, the selection of candidate genes was limited to genes known to be involved in the metabolism and toxicokinetics of naphthalene and PAHs and thus excluded other biological processes (e.g., cell proliferation and differentiation, cell signaling) that may be critical. Imputation from the HapMap panel (Collins 2009) could be applied to provide a route to testing additional SNPs without further genotyping; this would potentially improve the fine mapping resolution in candidate regions.

The *Sty*I array employed a SNP set based on linkage-disequilibrium patterns that supported approximately one-sixth genome coverage of the base-pair positions across all the chromosomes rather than coverage of known genes in particular pathways or networks. Thus, SNP assignment to genes and pathways is inherently difficult, but not impossible, in a reiterative approach and strategy as described here. In this context, the SNPs statistically associated with NKA level, as a polymorphic genetic biomarker, do not infer the functional basis for the SNP–phenotype association. Thus, genetic, epistatic, epigenetic, and/or noncoding RNA gene regulation interactions that may be associated with individual variation in NKA levels observed along with other predictors of naphthalene exposure are not excluded in establishing the predicted networks from the published curated data. Although the GWA results did not identify SNPs associated with NKA levels at the genome-wide significance level, three biologically plausible networks were predicted using SNPs identified by our strategy. An association test for a single SNP has limited utility and is insufficient to disclose the complex genetic structure of many intermediate phenotypes or diseases among naphthalene-exposed subjects. Diseases often arise from the joint action of multiple loci within a genetic structure or joint action of multiple genes within a pathway (Luo et al. 2010). Although each single SNP may be associated with only a small effect or disease risk, the joint action of multiple SNP-associated loci is likely to have significant risk effects. The predicted pathway and networks show that the identified SNP-associated genes are not independent but may interact with each other through biological processes that may contribute to the NKA levels. The significant SNP variants we identified are highly associated with the regulation of cellular processes and homeostasis and may contribute to differences in levels of targets for adduction by naphthalene electrophilic metabolites. Thus, we have shown that genetic variants and their potential role on biological functions may affect biomarker levels in naphthalene-exposed workers. These plausible genetic associations must be replicated and their biological functions validated.

In genome-wide association studies (GWAS), the genome-wide significance threshold is stringent, and few SNPs exceed the statistical requirement; genetic markers that do not equal or exceed this conservative threshold are generally ignored or neglected unless the biological plausibility is very strong. We used a high threshold level of genome-wide significance (i.e., 5.4 × 10^–7^) to protect against false-positive findings caused by multiple testing (O’Berg 1980; Risch and Merikangas 1996; Wolff et al. 1993). Meanwhile, it also necessitates careful consideration of the power to detect the effect size in the GWAS. For nearly all gene regions conclusively identified by GWAS, the per-allele effect sizes estimated are odds ratios < 1.5 (Hindorff et al. 2009). The variant alleles observed are usually common, and each allele is estimated to confer a small contribution to the overall effect. The mechanism by which these genetic variations affect the phenotype is not clear (Lee et al. 2006; Rioux et al. 2001; Yue and Moult 2006). By ranking the 10 SNPs with the lowest *p-*values for each NKA biomarker in PLINK, we were able to construct final exposure models and investigate the contributions of multiple SNPs using MLRM. Furthermore, analysis of predicted networks may minimize false-positive findings without missing important pathways due to a mandated stringent threshold. The statistical test for predicting networks, based on protein–protein or protein–gene interactions in the curated literature, treats each SNP/gene candidate as a sampling unit, instead of the individual within the sample population from which it was identified. Thus, the results are generalized to the sample of genes rather than to a new sample of individuals that may harbor those same alleles. Nonetheless, this approach provides a strategy for identifying and stratifying gene alleles for functional validation through molecular biology/reverse genetics and/or further sampling and testing in a other populations of exposed individuals.

Environmental factor contributions have been elusive in GWAS (Vineis et al. 2009). Further, the linear modeling framework in GWAS does not provide for robust analysis of the genomic and environmental factors (Moore et al. 2010), and there is no standard method for gene–environment interaction in GWAS. Identifying gene–environment interactions will be difficult in GWAS case–control studies given the paucity of exposure assessment in large multicenter epidemiological studies. Environmental exposure is more prevalent and is assessed with greater quantitative accuracy in a well-characterized sample population than in population or hospital-based case–control studies commonly used in GWAS performed to date (Vlaanderen et al. 2010). Both environmental and genetic component contributions may vary depending on the quantitative nature of the intermediate phenotype or disease. Identification of the significant contributing factors (environmental and genetic) to a phenotype is necessary to explain the population variance. Practically, we may only be able to determine the greatest size effects in small, well-defined populations. Genetic and epigenetic factors present in a population may have significant size effects that include heritable variation in traits that may not be directly associated with exposure. For example, heritable differences in blood pressure (essential hypertension) and respiratory rate and capacity may influence the absorption, distribution, and clearance of metabolites (biomarkers) and have effects greater than do xenobiotic metabolism and toxicokinetics in skin.

Allele frequency differences in an admixed population in an association study can bias results. Because this study population was not homogeneous, we investigated self-reported ethnicity to determine whether it contributes to skin NKA levels. The MLRM results showed that ethnicity was significant only for 2NKA and TNKA levels but not for the 1NKA level. Therefore, in the SNP association test, we controlled for ethnicity only for 2NKA and TNKA levels and not for the 1NKA level. We evaluated genome-wide and quantile–quantile plots of association results for individual SNPs with skin NKA levels [see Supplemental Material, Figures 1 and 2 (http://dx.doi.org/10.1289/ehp.1104304)]. We observed no significant deviation from the expected distribution of *p-*values of the tests for all SNPs. In addition, we performed principal component analysis and eigenvalue analysis (Patterson et al. 2006; Price et al. 2006), which showed that the population structure corresponded well with the self-reported ethnicity (see Supplemental Material, Figure 3). In the quantile–quantile plots, correlations approaching normality may hide substructures that confound outcome or, alternatively, deviations from normality may suggest substructure differences that may not actually exist, which can often be corrected (Voorman et al. 2011). We speculate that this apparent contradiction in quantile–quantile plots and principal component analysis may be due to our small sample population (i.e., low power) and/or a low-density SNP set based on physical coverage with low linkage disequilibrium.

The sample size for our study was very small compared with an average GWAS case–control study aimed to map variants associated with a disease. The small effect size of disease traits requires a large sample size to gain statistical power. This study population was well defined for the specific intrinsic and extrinsic factors measured. Furthermore, we used intermediate phenotypes rather than binary disease status, which is more objective (less misclassification) and more informative and can increase statistical power severalfold (Potkin et al. 2009).

In the models presented here, the number of parameters is likely to be large relative to the population sample size because we used the 10 SNPs with the lowest *p-*value for each intermediate phenotype (NKA level) to establish the final MLRM. Therefore, the *R*^2^ of the model may be increased. In addition, the naive estimators (from the model) of the effect size of the variants may overestimate the true effect size due to “winner’s curse” (Xiao and Boehnke 2011). In GWAS, this bias is increased by the use of stringent selection thresholds and ranking large numbers of SNPs. Genetic effect-size estimates are usually focused only on the variants showing significant evidence for an SNP–quantitative trait association. This may result in effect-size estimates that are upward biased (referred to as “ascertainment bias”); this is caused by ascertaining only those samples that result in genetic loci with evidence of significant association. Xiao and Boehnke (2011) showed that the winner’s curse affects the estimator of the coefficient of determination *R*^2^ in the context of quantitative association. Proportional bias of *R*^2^ was large when power was low, and it decreased when the power increased. Thus, the variation explained and the relative contribution of SNPs based on the *R*^2^ of the model may be inflated; therefore, our results should be interpreted with caution.

In summary, in a GWA with approximately 184,000 SNPs tested, 1,800 of these SNPs may be either false positive or false negative. By using multiple levels of testing for significance of association, we aimed to reduce the risk of selecting false positives by further testing groups of highly associated candidate genes by testing for gene–gene interactions in the curated literature that may lead to more refined analysis in replicate populations. We found three predicted networks that may be associated with these SNP variants and NKA biomarkers of exposure. The SNP variant associate genes and their products may be highly associated with *a*) regulation of cellular processes and homeostasis that alter the NKA biomarker levels, or *b*) adduction at cysteine residues by naphthalene metabolites. Also, another potential strategy is a two-stage design and independent replication of samples to confirm study findings (Collins 2009). However, replication is not easily achievable because populations are likely exposed to very different levels and mixtures of environmental toxicants (Vineis et al. 2009). Finally, the present study may be regarded as a discovery approach that can generate new hypotheses for plausible biological functions of potential genetic factors that affect exposure biomarker levels. Ultimately, only independent replication of these studies with increased sample size and/or functional validation of the candidate gene allelic variants will provide proof.

## Conclusions

We demonstrated a strategy to investigate the role of individual genetic variation in quantitative assessment of biomarkers of exposure in a well-characterized population of exposed individuals. Biomarker levels can be used as intermediate quantitative trait phenotypes when investigating association between dose–response relationship and individual exposure that may affect toxicity and disease outcomes. Thus, individual genome-wide variation should be accounted for in future genetic epidemiology and exposure assessment studies that use biomarkers of exposure and/or effect. This knowledge will *a*) increase our understanding of the exposure dose–effect relationships and improve exposure classification in epidemiology studies by reducing uncertainty in biomarker of exposure and/or early biological effect classification; *b*) help identify potential susceptible subpopulations with respect to exposure; and *c*) provide useful input in setting exposure limits by taking into account individual variations. Investigation of the predicted interactions between environment (extrinsic), individual genetic variation (intrinsic), and the biological outcome (phenotype) in occupational exposure studies as described here may provide an effective strategy and approach to identify human gene–environment interactions.

## Supplemental Material

(1.1 MB) PDF
