# Supplementary material for: Single-Nucleotide Polymorphisms Associated with Skin Naphthyl–Keratin Adduct Levels in Workers Exposed to Naphthalene
Source: Environ Health Perspect. 2012 Mar 5;120(6):857–64. doi: 10.1289/ehp.1104304 (PMC3385430; doi:10.1289/ehp.1104304)
Supplement: (1.1 MB) PDF [file ehp.1104304.s001.pdf]

Supplemental Material

Single Nucleotide Polymorphisms Associated with Skin Naphthyl-Keratin Adduct Levels in Workers Exposed to Naphthalene

R Jiang, JE French, VI Stober, J-CC Kang-Sickel, F Zou, and LA Nylander-French

Table of Contents

Supplemental Material: Methods

Genotyping, Allele Calls, and Data Cleaning........ 2

Supplemental Material: Tables

Supplemental Material, Table 1: Candidate genes (N = 35) and the number of SNPs (n = 498) tested for association with skin naphthyl-keratin adduct levels in fuel-cell maintenance workers using candidate-gene analysis........ 3

Supplemental Material, Table 2: Regression analyses of the significant covariates for the skin naphthyl-keratin adduct levels [ln(ng adduct/µg keratin)] in the fuel-cell maintenance workers (n = 100) exposed to jet fuel........ 4

Supplemental Material: Figures

Supplemental Material, Figure 1: Genome-wide plots for the associations between the $-\log_{10}P$ values of the skin naphthyl-keratin adduct levels controlled for covariates for 184153 SNPs and the SNP physical chromosome location........ 5

Supplemental Material, Figure 2: Quantile-quantile plots of genome-wide analyses of the observed versus expected $-\log_{10}P$ values........ 6

Supplemental Material, Figure 3: Principal Component Analysis (PCA) of the study population allelic variation and self-reported ethnicity........ 7

Supplemental Material: Discussion

Population Structure........ 8

Predicted Networks and Pathways Based upon Bioinformatic and MetaCore™ Analysis........ 8

References........ 10

Genotyping, Allele Calls, and Data Cleaning

Blood (10 ml) from each individual was collected into an EDTA tube (Vacutainer©; Becton-Dickinson and Company, Franklin Lakes, NJ). Samples were shipped overnight to the laboratory on Blue Ice© (Rubbermaid, Atlanta, GA) where stored at –70°C. Genomic DNA was isolated from 0.5 ml of whole blood using Puregene DNA extraction kit (Gentra, Minneapolis, MN) and quantified using a NanoDrop spectrophotometer (Thermo Fisher Scientific). Concentration and desalting of DNA was carried out by vacuum centrifuge (Savant Instruments Inc., Holbrook, NY) or ethanol precipitation, respectively. DNA (≥50 ng/µl, purity ≥1.8) was electrophoresed for quality control and processed for Sty*I* digestion and analysis using the GeneChip® Human Mapping 250k Sty*I* SNP array protocol (Affymetrix, Santa Clara, CA). In brief, 250 ng of DNA was digested using *StyI* restriction enzyme. An adaptor was ligated to the digested fragments and PCR performed using the added adapter sequences as primers. PCR products were cleaned and normalized prior to fragmentation and labeling with the fluorochrome for hybridization, washing, and image analysis.

Array images were acquired by scanning each array and raw DAT-image files were generated in GeneChip® Operating System (GCOS) software at UNC-CH Affymetrix Core. Each DAT image was processed by GCOS into CEL file and Affymetrix Genotyping Console 3.0.1 using BRLMM algorithm to genotype ≈238,000 SNPs for each individual. Each SNP genotype was generated as AA, AB, BB, or "no call" if the confidence value was less than 0.5.

Supplemental Material, Table 1. Candidate genes (N = 35) and the number of SNPs (n = 498) tested for association with skin naphthyl-keratin adduct levels in fuel-cell maintenance workers using candidate-gene analysis.

Gene	Number of SNPs	Gene	Number of SNPs	Gene	Number of SNPs
AHR	67	*CYP2A6*	4	*EPHX1*	4
AKR1C1	19	*CYP2B6*	4	*EPHX2*	19
AKR1C2	22	*CYP2C18*	1	*GSTM3*	2
AKR1C3	22	*CYP2C19*	9	*GSTM5*	2
AKR1C4	29	*CYP2C8*	10	*GSTT1*	1
ARNT	2	*CYP2C9*	9	*GSTT2*	6
ARNT2	47	*CYP2D6*	0	*NQO1*	1
CRABP1	8	*CYP2E1*	13	*SOD1*	7
CYP1A1	3	*CYP3A4*	1	*SOD2*	64
CYP1A2	29	*CYP3A5*	2	*SOD3*	18
CYP1B1	38	*CYP3A7*	3	*SRD5A2*	11
CYP26B1	51	*CYP4B1*	10		

Note: The total number of SNPs is 537 in this table, because some SNPs are shared by more than one gene, only 498 independent SNPs were tested on 35 genes.

Supplemental Material, Table 2. Regression analyses of the significant covariates for the skin naphthyl-keratin adduct levels [ln(ng adduct/µg keratin)] in the fuel-cell maintenance workers (n = 100) exposed to jet fuel.

Adducts	R^2	Predictor	Parameter Estimate	Standard Error	*P*-value
1NKA	0.271	Intercept	-0.416	0.285	0.147
		Dermal exposure	-0.033	0.019	0.083
		Age	-0.018	0.008	0.020
		Exposure time	0.002	0.001	0.013
		Replace foam	0.325	0.087	0.0003
		Smoking	0.132	0.076	0.086
2NKA	0.353	Intercept	0.633	0.239	0.010
		Dermal exposure	-0.052	0.016	0.001
		Age	-0.017	0.006	0.008
		Exposure time	0.004	0.001	<0.0001
		Replace foam	0.147	0.072	0.043
		Ethnicity	-0.173	0.094	0.068
TNKA	0.354	Intercept	0.941	0.234	0.0001
		Dermal exposure	-0.047	0.015	0.003
		Age	-0.017	0.006	0.007
		Exposure time	0.004	0.001	<0.0001
		Replace foam	0.186	0.070	0.009
		Ethnicity	-0.155	0.092	0.094

1NKA = 1-naphthyl-keratin adduct; 2NKA = 2-naphthyl-keartin adduct; TNKA = total naphthyl-keratin adducts.

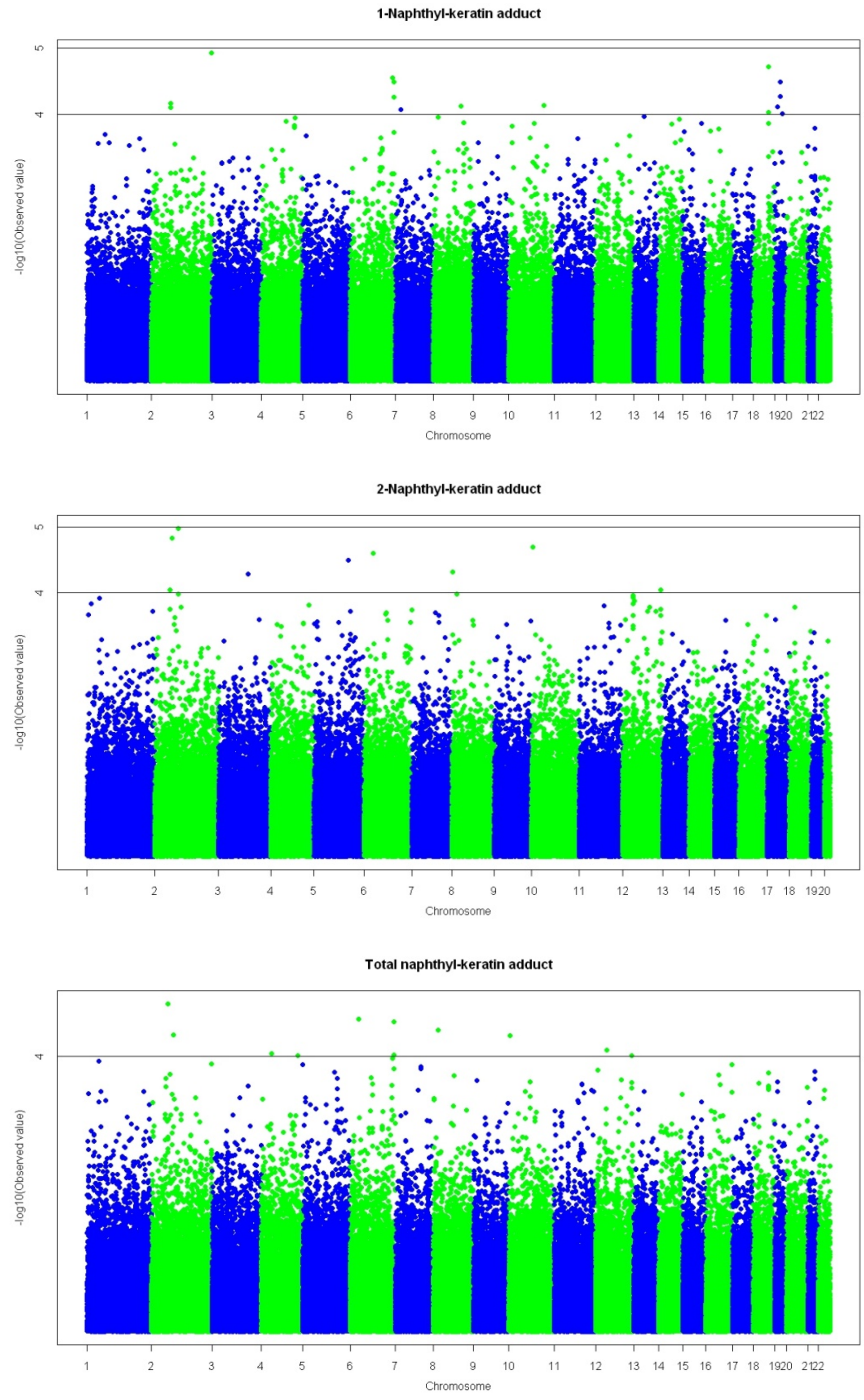

Supplemental Material, Figure 1. Genome-wide plots for the associations between the $-\log_{10}P$ values of the skin naphthyl-keratin adduct levels controlled for covariates for 184153 SNPs and the SNP physical chromosome location.

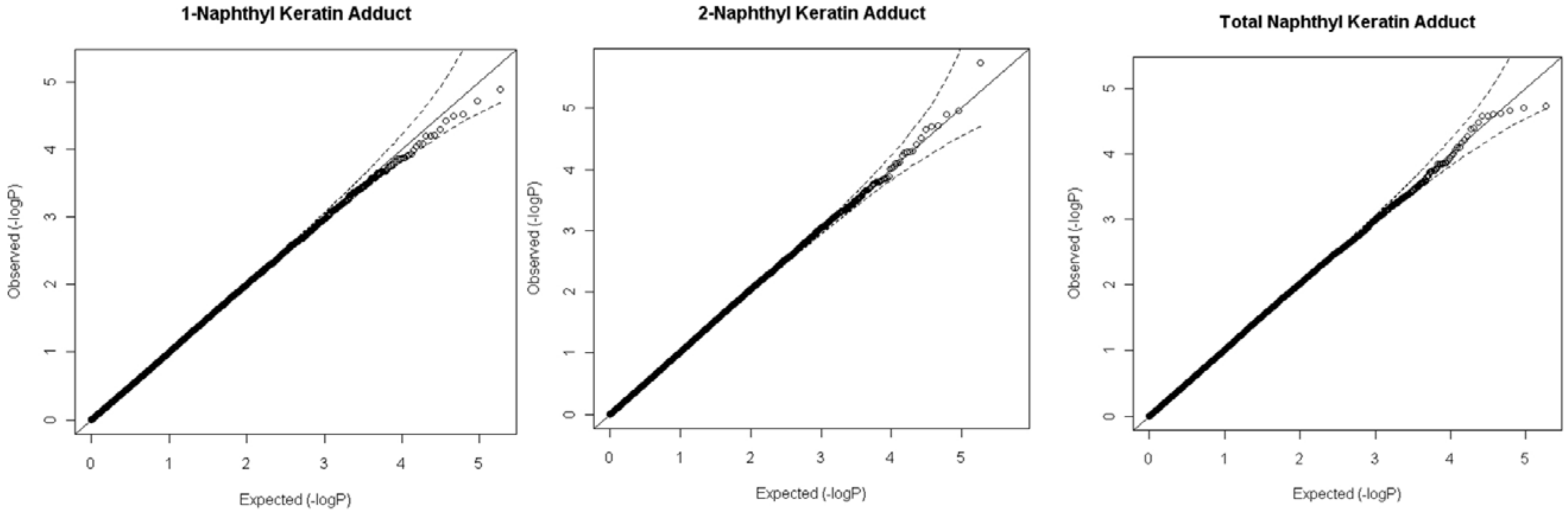

Supplemental Material, Figure 2. Quantile-quantile plots of genome-wide analyses of the observed versus expected $–\log_{10}P$ values. The results of the observed associations between the quantitative skin naphthyl-keratin adduct levels are plotted against the 184,153 SNPs and the expected distribution of the $–\log_{10}P$ under null hypothesis of no association (dash line 95% confidence level).

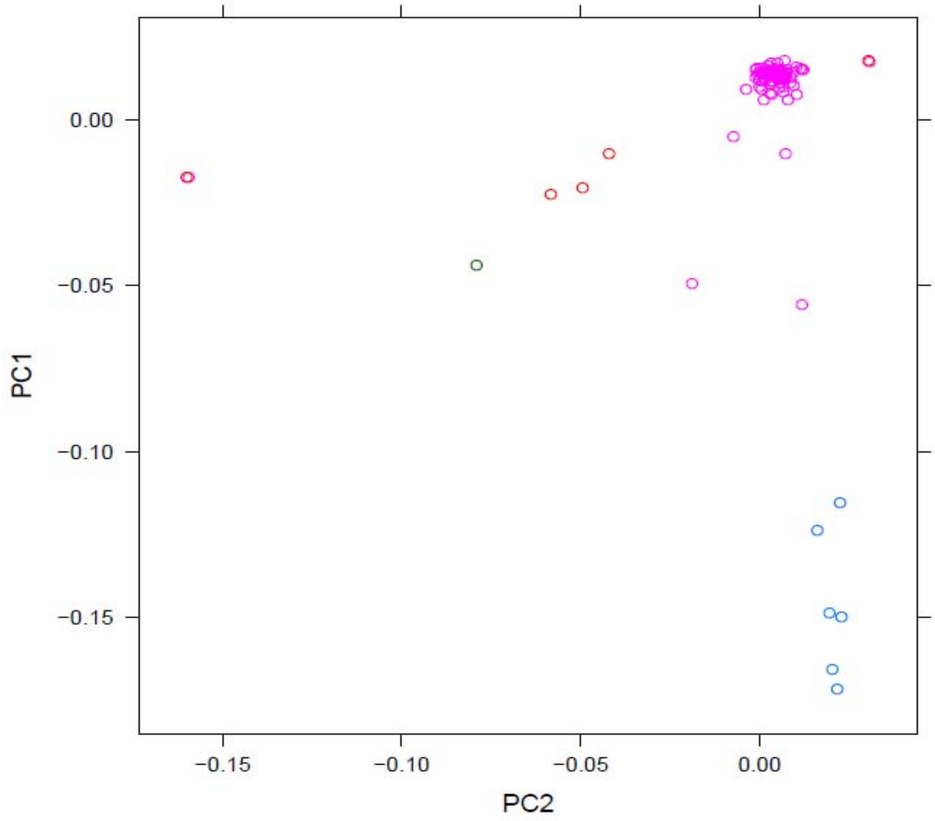

Supplemental Material, Figure 3. Principal Component Analysis (PCA) of the study population allelic variation and self-reported ethnicity (pink – Caucasian, blue – African American, red –Hispanic, and green – Asian).

Population Structure

Self-reported ethnicity for this study population indicated that population structure exists because one or more groups are included that likely have a distinct population genetic history. The SNP-NKA association quantile-quantile plot comparing different NKA biomarker levels shows that the observed versus expected p-values are in agreement until *P*-values approach significance and show a trend toward the observed –log*P* then deviate. At this point of deviation from the linear correlation, the null hypothesis would be rejected if the 95% confidence levels were exceeded. No indication of population structure was observed in the quantile-quantile plots for any of the SNP-NKA biomarker associations. However, correlations approaching normality may hide substructure issues that confound outcome or may suggest substructure differences that may not actually exist and that can often be corrected by adjustment to the regression models (Voorman et al. 2011).

Self-reported ethnicity may be biased without an examination of family history. PCA analysis was undertaken to compare individual SNP-allelic variation within the population with self-reported ethnicity (Supplemental Material; Figure 3). PCA of individual SNP variation and identification by self-reported ethnicity illustrates the observed structure within this population sample.

Predicted Networks and Pathways Based upon Bioinformatic and MetaCore™ Analysis

The first predicted network involves *CD47*, *RPS6KA2*, *KLF6*, and *MSRA* (Figure 1A), which are associated with the positive regulation of cellular and biological processes, chemical homeostasis, regulation of biological quality, and homeostasis ($P = 3.01 \times 10^{-19}$). *CD47* is an integrin-associated signal transducer, which is involved in the increase in intracellular calcium concentration that occurs upon cell adhesion to extracellular matrix. The encoded protein is also a receptor for the C-terminal cell-binding domain of thrombospondin, and it may play a role in membrane transport and signal transduction (Brown and Frazier 2001; Rebres et al. 2001).

KLF6 and *RPS6KA2* appear to interact within a predicted network that is strongly associated with the regulation of cell proliferation and differentiation, including keratinocytes and, thus, might influence skin NKA levels in the workers in this study. Promoter sequences of keratin-1 (KRT1) and keratin-10 (KRT10) target genes were used to identify KLF6 and CREB1 transcription factor motifs in the promoter region, which have not been published in the peer-reviewed literature. The motifs were predicted using Genomatix TransFac MatInspector (Cartharius et al. 2005). The KLF6 and CREB1 transcription factor binding site sequence identified on keratin-12 (Chiambaretta et al. 2002; Wang et al. 2002) was used to weight and develop a training set to interrogate similarity matrices and to identify predicted binding sites, which require functional studies for validation.

RPS6KA2 is affected by a mitogen-activated protein kinase (MAPK) and phosphorylates various substrates, including members of the MAPK signaling pathway. This kinase can phosphorylate CREB1, a transcription factor that both up-regulates and suppresses many other critical genes in cell proliferation and differentiation. The KLF6 transcription factor, a member

of the Krueppel-like family, can up-regulate KLF4 (which has overlapping functions with KLF6) and, along with the transcription factor CREB1, up-regulate corneal KRT12 (Chiambaretta et al. 2002; Wang et al. 2002). Bioinformatic analysis indicates that consensus transcription binding site sequences in KRT12 for both KLF6 and CREB1 transcription factor sites are predicted in KRT1 and KRT10 on or near their core promoter sequence. Multiple transcript variants encode different isoforms that have been found for this gene, some of which are implicated in carcinogenesis. KLF6 has also been described as a tumor suppressor gene (Miyaki et al. 2006; Narla et al. 2001; Reeves et al. 2004) and has been associated by epidemiologic genome-wide association studies (GWAS) with a number of diseases (Liu et al. 2010; Spinola et al. 2007). MSRA, a cytosolic methionine-*S*-sulfoxide reductase, carries out the enzymatic reduction of methionine sulfoxide to methionine and *MSRA* genes are differentially expressed in human skin (Taungjaruwinai et al. 2009). This enzyme functions to repair of oxidative damage to proteins and to restore biological activity. RA regulates *MSRA* expression via two promoters and three transcript variants encoding different isoforms have been observed that may alter function or tissue specificity (Pascual et al. 2009).

In the second predicted pathway, *ADD3*, along with *MSRA* and *CD47*, may also interact independently with *APOA1* and *CAV1* (Figure 1B) to regulate biological quality, homeostasis, cellular components of movement, positive regulation of biological process, and response to stimuli ($P = 2.09 \times 10^{-15}$). ADD3 is a member of a family of heteromeric proteins composed of different subunits referred to as Adducin alpha, beta and gamma. The three subunits are encoded by distinct genes and belong to a family of membrane skeletal proteins involved in the assembly of spectrin-actin network in erythrocytes and at sites of cell-cell contact in epithelial tissues (Joshi et al. 1991; Kaiser et al. 1989). *ADD3* has been identified in GWAS studies associated with genetic variation and blood pressure and hypertension phenotypes (Bianchi et al. 2005; Seidlerova et al. 2009). *CD47*, as described above, also encodes a membrane protein, which is involved in the increase in intracellular calcium concentration that occurs upon cell adhesion to extracellular matrix. The encoded protein is also a receptor for the C-terminal cell-binding domain of thrombospondin, which may play a role in membrane transport and signal transduction (Brown and Frazier 2001; Rebres et al. 2001). *APOA1* encodes the protein, apolipoprotein A-I, a major protein component of high-density lipoprotein (HDL) in plasma. This protein promotes cholesterol efflux from tissues to the liver and intestine for excretion. Two cysteine disulfide bonds are required for ABCA1 transport of APOA1 and are thus potential sites of adduction (Hozoji et al. 2009). *CAV1* encodes a scaffolding protein, which is the main component of the caveolae plasma membranes found in most cell types. This protein links integrin subunits to the tyrosine kinase FYN, an initiating step in coupling integrins to the RAS-ERK pathway and promoting cell cycle progression (Wary et al. 1998).

In the third pathway, *NRXN1* and *NRSN1* are associated with neuron differentiation, negative regulation of cell cycle, axon guidance, synapse assembly, and generation of neurons in the published literature (Figure 1C). NRXN1 and NRSN1 are associated NRXN1 (Neurexin 1) interacts with membranes and epidermal growth factor (EGF) through cysteine rich 6-laminin G-

like domain calcium mediated receptors and 1 calcium binding EGF domain, which also may be targets for electrophile adduction that affect ectodermal derived tissues. NRSN1 (Neurensin 1), a vesicular membrane protein, as well as NRXN1 and NLGN1 may be predicted to interact through the transcriptional factors ZNF217, AHR, and ESR1 and the kinases HDAC1 and HDAC2 through ubiquitin and the histone deacetylases (HDAC1 and HDAC2). NRSN1 and NRXN1 are integral to basement membranes of the nervous system (Lise and El-Husseini 2006) and may have functions in other ectodermal derived tissues not yet described. Both NRXN1 and NRSN1 expression is controlled by REST (RE1-silencing transcription factor, which is also a member of the Krueppel-type zinc finger transcriptional factor family like KLF6). We speculate that NRXN1, NRSN1, and NLGN1 have functions other than neuron synapse development or its tissue specific restrictions have been relaxed in the skin due to silencing of the tissue specific insulators or they may provide targets for adduction due to their cysteine rich protein structure. In the skin, we can speculate that tissue specific relaxation of expression might have an effect on membrane transport and/or epidermal keratinocyte differentiation.

References

Bianchi G, Ferrari P, Staessen JA. 2005. Adducin polymorphism: detection and impact on hypertension and related disorders. Hypertension 45(3): 331-340.

Brown EJ, Frazier WA. 2001. Integrin-associated protein (CD47) and its ligands. Trends Cell Biol 11(3): 130-135.

Cartharius K, Frech K, Grote K, Klocke B, Haltmeier M, Klingenhoff A, et al. 2005. MatInspector and beyond: promoter analysis based on transcription factor binding sites. Bioinformatics 21(13): 2933-2942.

Chiambaretta F, Blanchon L, Rabier B, Kao WW, Liu JJ, Dastugue B, et al. 2002. Regulation of corneal keratin-12 gene expression by the human Kruppel-like transcription factor 6. Invest Ophthalmol Vis Sci 43(11): 3422-3429.

Hozoji M, Kimura Y, Kioka N, Ueda K. 2009. Formation of two intramolecular disulfide bonds is necessary for ApoA-I-dependent cholesterol efflux mediated by ABCA1. J Biol Chem 284(17): 11293-11300.

Joshi R, Gilligan DM, Otto E, McLaughlin T, Bennett V. 1991. Primary structure and domain organization of human alpha and beta adducin. J Cell Biol 115(3): 665-675.

Kaiser HW, O'Keefe E, Bennett V. 1989. Adducin: Ca++-dependent association with sites of cell-cell contact. J Cell Biol 109(2): 557-569.

Lise MF, El-Husseini A. 2006. The neuroligin and neurexin families: from structure to function at the synapse. Cell Mol Life Sci 63(16): 1833-1849.

Liu CY, Wu MC, Chen F, Ter-Minassian M, Asomaning K, Zhai R, et al. 2010. A Large-scale genetic association study of esophageal adenocarcinoma risk. Carcinogenesis 31(7): 1259-1263.

Miyaki M, Yamaguchi T, Iijima T, Funata N, Mori T. 2006. Difference in the role of loss of heterozygosity at 10p15 (KLF6 locus) in colorectal carcinogenesis between sporadic and familial adenomatous polyposis and hereditary nonpolyposis colorectal cancer patients. Oncology 71(1-2): 131-135.

Narla G, Heath KE, Reeves HL, Li D, Giono LE, Kimmelman AC, et al. 2001. KLF6, a candidate tumor suppressor gene mutated in prostate cancer. Science 294(5551): 2563-2566.
Pascual I, Larrayoz IM, Rodriguez IR. 2009. Retinoic acid regulates the human methionine sulfoxide reductase A (MSRA) gene via two distinct promoters. Genomics 93(1): 62-71.
Rebres RA, Vaz LE, Green JM, Brown EJ. 2001. Normal ligand binding and signaling by CD47 (integrin-associated protein) requires a long range disulfide bond between the extracellular and membrane-spanning domains. J Biol Chem 276(37): 34607-34616.
Reeves HL, Narla G, Ogunbiyi O, Haq AI, Katz A, Benzeno S, et al. 2004. Kruppel-like factor 6 (KLF6) is a tumor-suppressor gene frequently inactivated in colorectal cancer. Gastroenterology 126(4): 1090-1103.
Seidlerova J, Staessen JA, Bochud M, Nawrot T, Casamassima N, Citterio L, et al. 2009. Arterial properties in relation to genetic variations in the adducin subunits in a white population. Am J Hypertens 22(1): 21-26.
Spinola M, Leoni VP, Galvan A, Korsching E, Conti B, Pastorino U, et al. 2007. Genome-wide single nucleotide polymorphism analysis of lung cancer risk detects the KLF6 gene. Cancer Lett 251(2): 311-316.
Taungjaruwinai WM, Bhawan J, Keady M, Thiele JJ. 2009. Differential expression of the antioxidant repair enzyme methionine sulfoxide reductase (MSRA and MSRB) in human skin. Am J Dermatopathol 31(5): 427-431.
Voorman A, Lumley T, McKnight B, Rice K. 2011. Behavior of QQ-plots and genomic control in studies of gene-environment interaction. PLoS One 6(5): e19416.
Wang IJ, Carlson EC, Liu CY, Kao CW, Hu FR, Kao WW. 2002. Cis-regulatory elements of the mouse Krt1.12 gene. Mol Vis 8: 94-101.
Wary KK, Mariotti A, Zurzolo C, Giancotti FG. 1998. A requirement for caveolin-1 and associated kinase Fyn in integrin signaling and anchorage-dependent cell growth. Cell 94(5): 625-634.
